# Design Strategies of Fluorescent Biosensors Based on Biological Macromolecular Receptors

**DOI:** 10.3390/s100201355

**Published:** 2010-02-12

**Authors:** Kazuki Tainaka, Reiko Sakaguchi, Hironori Hayashi, Shun Nakano, Fong Fong Liew, Takashi Morii

**Affiliations:** 1 Institute of Advanced Energy, Kyoto University, Uji, Kyoto 611-0011, Japan; E-Mails: tainaka@iae.kyoto-u.ac.jp (K.T.); reiko@iae.kyoto-u.ac.jp (R.S.); h.hiro@iae.kyoto-u.ac.jp (H.H.); snaka@iae.kyoto-u.ac.jp (S.N.); fong-fong@iae.kyoto-u.ac.jp (F.F.L.); 2 CREST, JST, Uji, Kyoto 611-0011, Japan

**Keywords:** design strategy of fluorescent biosensors, biological macromolecular receptor, genetically encoded fluorescent biosensors, chemically modified protein based sensors, signaling aptamers

## Abstract

Fluorescent biosensors to detect the *bona fide* events of biologically important molecules in living cells are increasingly demanded in the field of molecular cell biology. Recent advances in the development of fluorescent biosensors have made an outstanding contribution to elucidating not only the roles of individual biomolecules, but also the dynamic intracellular relationships between these molecules. However, rational design strategies of fluorescent biosensors are not as mature as they look. An insatiable request for the establishment of a more universal and versatile strategy continues to provide an attractive alternative, so-called modular strategy, which permits facile preparation of biosensors with tailored characteristics by a simple combination of a receptor and a signal transducer. This review describes an overview of the progress in design strategies of fluorescent biosensors, such as auto-fluorescent protein-based biosensors, protein-based biosensors covalently modified with synthetic fluorophores, and signaling aptamers, and highlights the insight into how a given receptor is converted to a fluorescent biosensor. Furthermore, we will demonstrate a significance of the modular strategy for the sensor design.

## 1. Introduction

Molecular tools for shedding light on the complex interplay between macromolecules, signaling molecules, and biologically important ions inside the cells play a central role in molecular and cell biology. Much attention has been devoted by chemists and biologists for the past two decades to develop a “biosensor” that allows the real-time tracking of a small molecule of interest in living cells. A biosensor consists of a receptor component to capture a target ligand and a signal transduction component to convert the ligand-binding event into measurable signals, such as fluorescence, chemiluminescence, colorimetric, electrochemical, and magnetic responses. Especially, fluorescence detection is currently the most widely utilized method in the biomolecular imaging due to its high sensitivity and selectivity, sufficient temporal and spatial resolution, and low cost for use [1–5]. In this review, we focus on the biosensor detecting an analyte of interest by means of fluorescence signals owing to limitations of space.

Various kinds of fluorescent biosensors constructed by synthetic receptors [6–13] and biological macromolecular receptors such as proteins [5,14] and aptamers [15] have been reported to date. Although we appreciate the contribution of synthetic fluorescent sensors to our understanding of biochemical activities in living cells [7–9], the scope of this review is limited to overview the design of fluorescent biosensors composed of biological macromolecular receptors. The construction of fluorescent biosensors generally relies on the rational design strategy as follows. The first step requires an effort to find a macromolecular receptor with appropriate affinity and specificity to the target. The second step integrates the signal transduction function induced by the molecular recognition event into the receptor. Because the native biological receptor usually lacks an inherent property of the signal transduction function, foreign reporter moieties such as an auto-fluorescent protein (AFP) and a synthetic fluorophore must be introduced at the appropriate position of the receptor component. In spite of a seemingly simple procedure, researchers attempting to fabricate a novel fluorescent biosensor for a given target would inevitably struggle with unexpected labours. Adoption of the previously established methodologies would enable us to escape from most of the difficulties. This review presents a brief survey of a variety of design strategies for fluorescent biosensors with an emphasis on the guiding principle. Apart from the conventional design strategy, a unique modular strategy for tailoring fluorescence biosensors by a simple combination of a receptor and a signal transducer has recently been proposed in the field of signaling aptamers [16,17]. We will discuss the advantage of the strategy and will refer to the perspective of fluorescent biosensors based on macromolecular receptors.

## 2. Auto-Fluorescent Protein (AFP) Based Biosensors

Auto-fluorescent proteins (AFPs) such as green fluorescent protein (GFP) from the jellyfish [18] are well-established and versatile reporter proteins for monitoring gene expression profiles [19] and protein localizations [20] in a variety of systems. It is noteworthy that AFPs exhibit spontaneous fluorescence emission in cells by the autocatalytic formation of the chromophore after translation [21,22]. Therefore, AFPs can be endogenously expressed in cells or tissues just by transfection of the plasmid DNA without interfering with their fluorescence properties and damaging the cells. In addition to the application of AFPs as a reporter tag, various kinds of AFP-based biosensors have recently been developed by fusion of receptor proteins or mutation of AFPs. There are practically two strategies for the construction of AFP-based biosensors; (a) analyte-sensitive sensors and, (b) conformation-sensitive sensors [23].

The design of analyte-sensitive sensors was based on AFP variants, whose fluorescent properties were directly affected by the interaction between a target molecule and a chromophore moiety in AFP. Initially, pH and halide-sensitive AFP variants have been developed exploiting the intrinsic pH sensitivity of GFP mutants [24–26] and the high p*K*_a_s of YFP mutants [27–29]. Mutations in close proximity to the GFP chromophore or the barrel structure of BFP lead to the specific biosensor for Hg^2+^ [30] or Zn^2+^ [31], respectively. In this type of sensor, the receptor function was directly integrated into the chromophore itself by the alteration of the chemical nature around the chromophore.

The conformation-sensitive sensors are designed so that the conformational change of the receptor associated with the ligand-binding event transduces to a significant fluorescence response of AFPs. This design strategy is more versatile than that for the analyte-sensitive sensor, because this type of sensor could be applied to a variety of native receptor proteins. Actually, biosensors for Ca^2+^ [32–43] as well as for small organic molecules such as ATP [44], cAMP [45–48], cGMP [49–51], tryptophan [52], glutamate [53,54], and inositol phosphates [55,56] have been reported based on this strategy. This type of sensors could be classified in three categories; (1) dual AFP-fused fluorescent resonance energy transfer (FRET)-based biosensors, (2) single circularly permuted (cp) AFP-based biosensors, and (3) split AFP-based biosensors. In this section, we will describe these strategies in more detail, highlighting the insights from the practical standpoint.

### 2.1. Dual AFP-Fused FRET-Based Biosensors

**Figure 1.**
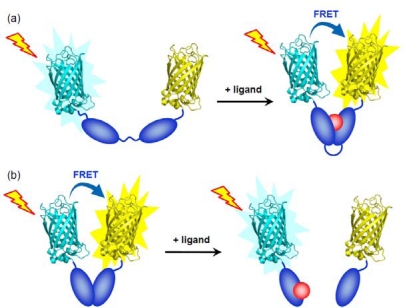
Schematic illustration shows a concept of ligand sensing by dual AFP-fused FRET-based biosensors. Currently, CFP and YFP mutants are preferentially selected as FRET donor and acceptor, respectively. (a) Intramolecular FRET-based biosensors exploit the protein domains with a large structural change upon the ligand-binding event. (b) Intermolecular FRET-based biosensors accompany the dissociation or association of multiple subunits upon the ligand-binding event. The dissociation-type FRET-based biosensor is only depicted in this figure.

FRET is a physicochemical phenomenon which only occurs when two fluorophores are in sufficient proximity (<10 nm) of each other, and the emission spectrum of the donor overlaps the excitation spectrum of the acceptor [57]. In the AFP-based FRET strategy, CFP and YFP mutants have been favorably utilized as a FRET donor and an acceptor, respectively [58]. Engineering with two AFPs in combination with the receptor protein affords a sensor protein that responds to dynamic fluctuation of intracellular ligand concentration by a ratiometric fluorescence change. The feasibility of this strategy strongly depends on the magnitude of the structural change of the receptor. In the case of the receptor with a large structural change upon binding the substrate, this strategy would be the most straightforward way to integrate the signal transduction function into the receptor of interest (Figure 1a), although serious concerns have been pointed out that the obtained FRET signals do not simply reflect the change in the expected distance of FRET pairs [58,59]. The trailblazing work for this sensor was reported by Miyawaki *et al*., in which a genetically encoded calcium indicator composed of two different colored AFP mutants and calmodulin, a Ca^2+^ receptor, has been constructed [36].

The receptor complex that accompanies the dissociation or the association of multiple subunits upon ligand binding was also suitable for the design of FRET-based biosensors (Figure 1b). Zaccoro and coworkers constructed a ratiometric fluorescent biosensor for cyclic adenosine monophosphate (cAMP) on the basis of an intermolecular FRET system between regulatory (R) and catalytic (C) subunit of protein kinase A (PKA) [47,48]. This biosensor can detect the rise of intracellular cAMP concentration by the decrease in the FRET efficiency induced by the dissociation of C subunit from R subunit. More comprehensive information on dual AFP-fused FRET-based biosensors is available in other excellent reviews [60–62].

Recently, Johnsson and coworkers have devised another class of FRET-based semisynthetic biosensors for Zn^2+^ combined with an AFP and a synthetic fluorophore based on the SNAP-tag labeling technology [63]. Because SNAP-tag fusion protein can be covalently modified with *O*^6^-benzylguanine derivatives in living cells [64], the approach has a potential for *in situ* preparation of semisynthetic fluorescent biosensors even in living cells.

### 2.2. Single Circularly Permuted (cp) AFP-Based Biosensors

As an alternative strategy, non-FRET biosensors based on a single circularly permuted (cp) AFP have been developed for targeting Ca^2+^ [38–40,43], cGMP [65], H_2_O_2_ [66,67], Zn^2+^ [68], and an inositol phosphate derivative [56]. cpAFPs were constructed by connecting original N and C termini by a short peptide linker and regenerating the novel N and C termini at a specific position. A number of cpAFPs with novel termini retained their fluorescence even when a foreign receptor was inserted into the termini [40]. Nakai and coworkers reported a high affinity and signal-to-noise Ca^2+^ indicator (termed as G-CaMP) composed of a single cpGFP and calmodulin [38]. Since G-CaMP responded fast enough to track the dynamic change in Ca^2+^ concentration, this sensor was a powerful tool for visualizing the intracellular dynamics of Ca^2+^ in living cells.

**Figure 2.**
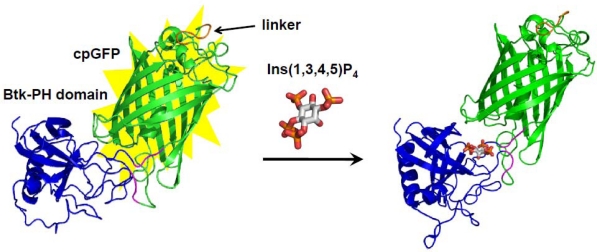
Schematic illustration shows a fluorescent biosensor for inositol tetrakisphosphate based on the split Btk PH domain-cpGFP conjugate [56]. The original N and C termini are linked with a short peptide linker (orange), and the novel terminal of cpGFP (purple) is fused to the split Btk PH domain (blue). The conformational change of the PH domain induced by the ligand-binding event was transduced to the structural perturbation at the chromophore of conjugated GFP, and then resulted in the ratiometric fluorescence change of cpGFP.

Although the dual AFP-fused FRET-based biosensor is the most facile and robust strategy among AFP-based biosensors, the application of the strategy for FRET-based sensors is essentially difficult in the case of a receptor that undergoes just a slight structural change upon binding to the substrate. In such a case, a sophisticated manipulation of the interplay between the small perturbation of the receptor conformation and the alteration of photochemical property of the AFP chromophore in the ligand-binding event must be required. In the cpAFP-based biosensors, the receptor protein could be placed at the residues near the chromophore that would critically affect the photochemical property of the chromophore in AFP. Morii and coworkers developed a novel cpAFP-based sensor for d-*myo*-inositol-1,3,4,5-tetrakisphosphate (Ins (1,3,4,5) P_4_), from a newly designed split pleckstrin homology (PH) domain of Bruton’s tyrosine kinase (Btk) and a single cpGFP [56] (Figure 2). The resulting split PH domain-cpGFP conjugate, Btk-cpGFP, exhibited bimodal absorption spectra corresponding to the protonated and deprotonated states of the chromophore in GFP. Interestingly, the Btk-cpGFP realized a ratiometric fluorescence detection of Ins (1,3,4,5) P_4_ by the excitation of each distinct absorption band, and retained the ligand affinity and the selectivity of the original PH domain.

### 2.3. Split AFP-Based Biosensors

A major drawback in the above-mentioned two strategies would be the modest fluorescence response upon the ligand binding due to the unavoidable background fluorescence of AFPs. In order to accomplish full suppression of the initial fluorescence, an AFP variant was divided into two fragments. A split GFP displayed quite a low background fluorescence in the separated state and recovered a fluorescence emission significantly by the reassembly of the two fragments when they were attracted in close proximity by a foreign driving force [69]. Based on this split GFP strategy, a receptor composed of two subunits that are associated by binding to the analyte can be converted into a fluorescent biosensor by connecting each of the two subunits with each split GFP fragment. A variety of fluorescent biosensors for detecting Ca^2+^ [43], specific DNA sequences [70,71], DNA methylation [72], and mRNA [73,74], have been constructed. Ghosh and coworkers have devised a split GFP based biosensor for the sequence-specific detection of DNA-methylation at CpG dinucleotides [72]. This sensor consisted of two fragments of the GFP-receptor fusion protein. One GFP fragment was attached by a zinc-finger protein targeting a specific sequence of double stranded DNA, while the other complementary GFP fragment was attached by a methyl-CpG binding domain protein targeting an adjacent methylated CpG dinucleotide site. In the presence of both DNA sites, the reassembly and concomitant fluorescence recovery of the reconstituted GFP was occurred.

## 3. Chemically-Modified Protein Based Sensors

Another strategy for constructing fluorescent biosensors is a structure-based design of a protein-based biosensor covalently modified with a synthetic fluorophore. Genetically encoded biosensors using AFPs can be expressed basically in all types of cells, allowing straightforward visualization of intracellular target molecules. In contrast, these protein-based biosensors require the invasive technique for translocating across the plasma membrane, such as electroporation [75–77], lipofection [78,79], microinjection [80], and tagging arginine-rich sequences [81,82]. However, this type of biosensors could be more advantageous in some respects. The relatively smaller size of the synthetic fluorophore is likely to contribute to the less perturbation of the property of the original receptor protein. Furthermore, since the amount of intracellular biosensors can be controlled easily compared to the genetic expression system, the interference to the molecular geography of the analyte in cells could be suppressed minimally. Thus, the combination of these two types of biosensors would prompt us to illuminate the *bona fide* picture of the analyte in cells.

In the synthetic fluorophore-attached biosensors, the principle of the signal transduction mechanism is generally based on the alteration of microenvironment of the fluorophore during the ligand-binding event. Especially, polarity-sensitive fluorescent probes are the most widely utilized due to the abrupt change in hydrophobicity in the vicinity of the protein surface [5]. The central issue for the construction of these biosensors is the way to introduce the fluorophore into the receptor protein site-selectively. Current methodologies for the site-selective incorporation of synthetic fluorophores into protein are divided into three groups; (1) incorporation of a thiol reactive fluorophore by engineering of a mutant receptor with a unique cysteine residue, (2) a site-specific unnatural amino acid mutagenesis with an expanded genetic code, and (3) a post-photoaffinity labeling modification.

### 3.1. Incorporation of a Thiol Reactive Fluorophore by Engineering of a Mutant Receptor with a Unique Cysteine Residue

In this method, all of the original cysteine residues must be initially substituted with other amino acids followed by the introduction of a unique cysteine residue at the specific position to avoid the non-specific labeling. The incorporation site of a fluorophore could be determined by the three-dimensional structure of the receptor protein obtained from crystallographic analysis.

**Figure 3.**
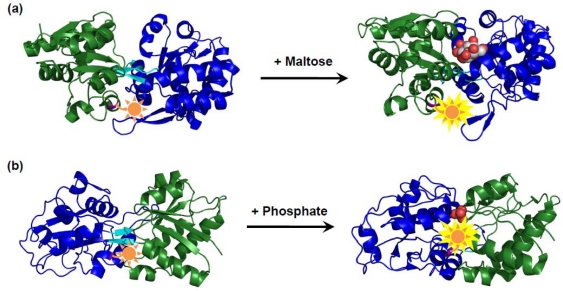
Schematic illustrations show fluorescent biosensors for (a) maltose [84] and (b) inorganic phosphate [93]. The maltose-binding protein (MBP) and phosphate-binding protein (PBP) change from an apo state (left) to a liganded state (right) by a bending-twisting motion of the N domain (green) and C domain (blue) about the hinge region (cyan). A fluorescent reporter group (orange) to monitor ligand binding has been attached to Asp 95 in MBP or Ala 197 in PBP, where was identified as an allosteric site or a peristeric site, respectively. The residues are indicated in magenta.

Hellinga and coworkers have developed a variety of synthetic fluorophore modified biosensors based on *E. coli.* periplasmic maltose-binding protein (MBP) families [83]. MBPs consist of two domains connected by a hinge region, with a ligand-binding site located at the interface between the two domains, which can adopt two different conformations. The regions induced allosteric conformation changes in response to the ligand binding was identified in MBPs. Environmentally sensitive fluorophores were covalently attached to unique thiols introduced by cysteine mutations within these regions to create biosensors for maltose [84] (Figure 3(a)), glucose [85], ribose [86], and glutamine [86]. Furthermore, extension of the natural diversity by a computational design has provided allosteric fluorescent biosensors for Zn^2+^ [87,88], trinitrotoluene [89], L-lactate [89], serotonin [89], and pinacolyl methyl phosphonic acid [90]. However, since the predicted conformational changes upon substrate binding from the computational design does not necessarily coincide with the conformational changes revealed by the crystal structure, the efficacy of computational design of ligand binding remains controversial [91,92].

Apart from the attachment of fluorophores to allosteric sites of MBP, other group demonstrated that introduction of a fluorophore onto the binding pocket of MBP has also provided a fluorescent biosensor for maltose [94]. The A197C mutant of *E. coli.* phosphate-binding protein (PBP) was also successfully converted into a fluorescent biosensor for inorganic phosphate (P_i_) by the conjugation of thiol-specific coumarin maleimide to Cys197, which is located at the periphery of the binding site [93,95] (Figure 3(b)). The significant fluorescence enhancement of the coumarin-labeled PBP upon binding P_i_ was discussed with regard to a specific interaction of the coumarin fluorophore with e protein based on the crystallographic analysis [96]. Its rapid response and high selectivity to P_i_ were suitable for monitoring changes of the P_i_ concentration in real time [93,97].

**Figure 4.**
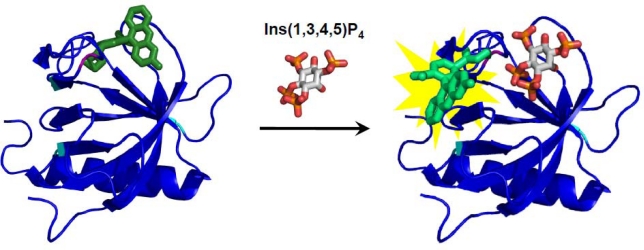
Schematic illustration shows a fluorescent biosensor for Ins (1,3,4,5) P_4_ based on the GRP1 PH domain covalently modified with a fluorescein (green) as a reporter probe [77]. The original cysteine residues (cyan) were replaced with other amino acids. The fluorophore was designed to be oriented near the binding pocket. The position labeled by the fluorescein at Glu 82 is indicated in magenta. The local environmental change of the fluorophore induced by the ligand-binding event was transduced to the fluorescence change.

Morii and coworkers constructed novel biosensors for inositol 1,4,5-trisphosphate (Ins (1,4,5) P_3_) and 1,3,4,5-tetrakisphosphate (Ins (1,3,4,5) P_4_) by utilizing the pleckstrin homology (PH) domain of phospholipase C (PLC) δ_1_ [98] and the general receptor for phosphoinositides 1 (GRP1) [77] (Figure 4), respectively. In these biosensors, a synthetic fluorophore was attached at the proximity of the ligand-binding site so that the changes in orientation of the fluorophore induced by the substrate binding leads to a sufficient fluorescence response. This structure-based design of synthetic fluorophore-modified biosensors is a powerful method to produce biosensors with high selectivity and appropriate affinity to target inositol derivatives in living cells [77,82,99].

A class A *β*-lactamase that can efficiently bind to and hydrolyze *β*-lactam antibiotics has been altered into a fluorescent biosensor for those antibiotics [100,101]. Replacement of the catalytically important residue with a cysteine following a fluorophore-labeling drastically suppressed the enzymatic activity, but instead allowed fluorescence “turn-on” sensing of substrates. Because the biosensor showed a significant fluorescent response to a large variety of *β*-lactam antibiotics, it can be a promising tool for the discovery of new *β*-lactamase inhibitors.

### 3.2. Site-Specific Unnatural Amino Acid Mutagenesis with an Expanded Genetic Code

Unlike the post-labeling of unique cysteine residues, a mutagenesis technique for direct incorporation of synthetic fluorophores as unnatural amino acids into desired positions in proteins has been developed. Such a site-specific mutagenesis with an expanded genetic code that employed an amber suppression method [102,103] or a four-base codon method [104] in cell-free translation systems has provided a variety of fluorescently modified proteins [105–107].

Hohsaka and coworkers synthesized unnatural amino acids modified with BODIPY derivatives and incorporated two of them into different positions of calmodulin as a donor and acceptor pair for FRET using two four-base codons [107]. The doubly modified calmodulin exhibited a substantial FRET signal in response to the conformational change of calmodulins induced by the addition of the calmodulin binding peptide.

### 3.3. Post-Photoaffinity Labeling Modification

When a three dimensional structure of a receptor protein is not available, it is difficult to convert such a receptor into a fluorescent biosensor by applying the above mentioned methods. An approach enabling a site-specific incorporation of a signal transducer proximal to the binding pocket of intact protein, for which little or no structural information is available, is also highly desirable.

Hamachi and coworkers have reported a new method, which was termed as post-photoaffinity labeling modification (P-PALM) [108], to incorporate a unique benzyl thiol group in the vicinity of the sugar-binding pocket of concavalin A (ConA) that is a well-studied lectin. Incorporation of a thiol group to the ligand-binding pocket of the receptor is conducted by a photoaffinity labeling molecule, which is composed of a ligand moiety, a photoreactive group and a cleavable disulfide group. Subsequently, conjugation of a thiol reactive fluorophore in that site affords a various types of fluorescent biosensors for specific saccharides [109–113]. Recently, the authors have expanded the P-PALM strategy to develop a ligand-directed tosyl (LDT) chemistry-based approach [114,115]. A detailed description of the P-PALM and the LDT strategies is found in other excellent review articles [5,108].

## 4. Signaling Aptamers

Combinatorial chemistry with *in vitro* evolution, known as SELEX (systematic evolution of ligands by exponential enrichment), offers an effective strategy for generating DNA or RNA receptors (aptamers) with appropriate affinity and specificity for various targets ranging from small molecules to proteins [116–120]. Because most of the structurally characterized aptamers undergo induced-fit type conformational changes upon binding of their cognate ligands [121], the introduction of signal transduction function can be accomplished by taking advantages of the ligand dependent change in the local environment around the attached fluorophores. Although the design strategy of fluorescent biosensors based on the preceding protein receptor is the most practical approach at present, application of this strategy is limited by the availability of proteins as a receptor for the targets. Even if there exists no report of a specific or an appropriate protein receptor for the substrate of interest, aptamers for the substrate can potentially be generated through *in vitro* selection. In this respect, the design of aptamer-based fluorescent sensors represents an attractive and promising alternative to the protein-based sensors. Additionally, development of the various types of fluorescence-signaling aptamers has been accelerated by the facile preparation of aptamers modified with synthetic fluorophores in a site-selective manner. In this section, we put emphasis on the method to integrate the signal transduction function into the aptamer apart from mentioning the selection and evolution techniques in detail. Until now, (1) covalently modified aptamer sensors and (2) dual fluorophore-labeled FRET-based aptamer sensors (termed as aptamer beacon) have been developed by following strategies described for the protein-based fluorescent biosensor. Significantly, unique modular strategies for tailoring aptamer sensors have been reported, which would relieve the lengthy and laborious trial-and-error to construct a sensor with an optimized function. Further information about the conventional types of aptamer sensors has recently been reviewed elsewhere [15,122].

### 4.1. Aptamer Sensors Covalently Modified with Synthetic Fluorophores

A signaling aptamer covalently modified with a synthetic fluorophore was first explored through a structure-based design by Ellington and co-workers [123]. Similar to the design of protein-based biosensors, a fluorescent reporter was initially placed either in proximity to the ligand-binding site or at the relatively movable position during the ligand-binding event. When acridine or fluoroscein as a signal transducer was introduced close to the binding pocket of anti-ATP RNA or DNA aptamers, respectively, fluorescence emissions of these aptamers were responded to the ATP concentration. The researchers also proposed a direct selection strategy of the signaling aptamers from randomized nucleic acid sequence libraries containing fluorescent nucleotides [124]. A bis-pyrene fluorophore, whose excimer and monomer emission are highly susceptible to the local structural change, was conjugated with an anti-ATP DNA aptamer by Yamana and co-workers [125]. This fluorescent aptamer displayed an efficient ratiometric fluorescence response toward ATP concentration. The electrostatic environment near the 2′-ribose position in nucleic acids highly depends on the local conformational constraint of the nucleotide due to the electrostatic interaction with the adjacent 3′-phosphodiester backbone group [126,127]. Anti-ATP DNA aptamers involving the 2′-pyrene-modified nucleoside devised by the same group served as a fluorescent biosensor with high affinity for ATP [128]. Weeks and coworkers engineered three kinds of RNA aptamer sensors for AMP, tyrosinamide, and argininamide, containing a 2′-BODIPY-modified nucleotides [129]. Furthermore, the researchers demonstrated that this type of sensors could be applicable to the aptamer without any structural information. As another candidate, fluorescent nucleotide analogues such as 2-aminopurine (2AP), 4-amino-6-methylpteridone (6MAP), and 3-methylisoxanthopterin (3MI), were also able to perform efficient signal transduction in aptamer sensors. Katilius and coworkers converted aptamers for human α-thrombin, immunoglobulin E, and platelet-derived growth factor B into the fluorescent biosensors for each targets by utilizing fluorescent nucleotide analogues [130]. The thrombin-binding aptamer with 6MAP showed a 30-fold thrombin-induced fluorescence enhancement.

### 4.2. Dual Fluorophore-Labeled Aptamer Sensors

Analogous to the case of protein-based biosensors, aptamers accompanied by the drastic conformational change upon ligand binding can be altered to biosensors that transduce the ligand binding event into the ratiometric FRET signal by attaching donor and acceptor pairs at the two ends. Takenaka and coworkers employed the formation of a G-quadruplex structure induced by K^+^ as a ratiometric fluorescent sensor for K^+^ [131,132]. By replacement of FRET pairs with a fluorophore and a corresponding quencher, this type of aptamers can be also adapted to the molecular beacon type of signaling method that allows a facile modulation of the fluorescence intensity. Such signaling aptamers known as aptamer beacons are believed to produce a comparatively larger fluorescence signal change than the rational designed aptamer sensors. The aptamer beacon approach has produced a variety of fluorescent biosensors for metal ions [133], small molecules [134–136], and proteins [137–139]. Besides the aptamer beacon composed of single architecture, split aptamer beacons combined with multiple aptamer fragments have also been constructed [140–143]. Li and coworkers reported a structure switching aptamer consisting of three fragments; a template DNA involving aptamer sequence and two small antisense strands bearing a fluorophore or a quencher [142]. This aptamer sensor exploits the target-induced switching between a DNA/DNA duplex and a DNA/target complex. In the absence of the analyte, the emission from the fluorophore was reduced by the quencher because the fluorophore and the quencher were designed that brought close to each other in DNA/DNA duplex form. Addition of the analyte causes the release of the antisense strand with the quencher and the significant increase of fluorescence intensity.

### 4.3. Modular Strategies for Tailoring Aptamer Sensors

The rational design strategy that has successfully provided fluorescent biosensors including not only the protein-based biosensor but also the aptamer-based biosensor appears to be the most convenient approach for the construction of biosensors. However, this strategy usually requires the redundant optimization of sensor functions because the introduction of fluorophore moieties frequently impairs the original receptor function and does not always guarantee to execute the expected optical signals. Furthermore, since the interplay between the molecular recognition event and the signal-transduction function is essentially unique to the individually constructed biosensor, it is also quite difficult to apply empirically obtained findings from the construction of a biosensor to the other biosensors. A modular strategy that permits facile preparation of biosensors with tailored characteristics by a simple combination of a receptor and a signal transducer has recently emerged as a new paradigm for a versatile design of fluorescent biosensors. Stojanovic and coworkers have reported a modular design of signaling aptamers based on the allosteric regulation of binding events [16]. These chimeric aptamers composed of two modular aptamers, one for the target recognition and another for holding a reporter dye, displayed robust fluorescence responses to three different targets.

**Figure 5.**
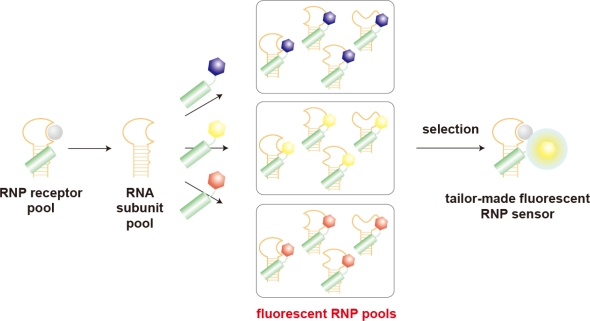
Schematic illustration shows a screening strategy of a tailor-made RNP fluorescent sensor [17]. Combination of the RNA subunit library of the RNP receptor and several fluorophore-labeled Rev peptide subunits generates combinatorial fluorescent RNP receptor libraries, from which RNP sensors with desired optical and/or binding properties are screened.

Morii and coworkers have recently developed a conceptually new strategy for simultaneous preparation of fluorescent biosensors with diverse functions based on a framework of ribonucleopeptide (RNP) [17] (Figure 5). In the first step to construct the fluorescent RNP sensor, a structure-based design based on the Rev Responsive Element (RRE)-HIV Rev peptide complex affords an RNA-derived RNP library by introducing a randomized nucleotides region as a ligand binding domain adjacent to the RRE segment. *In vitro* selection method was applied to the RNA-derived RNP library to afford a series of RNP receptors for a given target [144]. In the second step, by the modification of the Rev peptide subunit of the RNP receptor with a fluorophore, the RNP receptor is converted to a fluorescent RNP complex that showed the fluorescent intensity changing upon binding to the target molecule without losing the affinity and the selectivity of parent RNP receptor. The RNP receptors obtained by *in vitro* selection have a variety of RNA structures and reveal different affinity to the target molecule, and therefore are considered as an RNP receptor library. On the other hand, the peptide subunit is easily converted to a fluorophore-modified Rev peptide library by modifying chromophores with a variety of excitation and emission wavelengths. By taking the advantage of the noncovalent nature of the RNP complex, RNP sensors with desired optical sensing properties can be selected in a high-throughput manner by combining a series of RNA subunits derived from the RNP receptor library and a fluorophore-modified Rev peptide library. Actually, the modular strategy has produced a variety of fluorescent biosensors for targeting ATP [17], GTP [17], histamine [145], phosphotyrosine (pY) [146], and even a peptide fragment containing the pY residue within a defined amino acid sequence [147].

Though the strategy conveniently provides fluorescent RNP sensors, the noncovalent configuration becomes a disadvantage for the practical measurements, for instance, when the sensor concentration is reduced below the nanomolar range where the RNP complex would dissociate to each component. A covalently linking of the RNA and the peptide subunits without sacrificing the sensing function would overcome such disadvantages.

## 5. Perspective

Currently, the protein-based biosensors represent the most practical and reliable tools for the real-time measurements of various biologically important molecules in living cells, and they have actually contributed significantly to elucidate the function of those molecules in the cell. However, the fact that there exists a wide variety of design strategies for the protein-based biosensors inversely reflects a lack of a universal approach for the integration of the signal transduction function into the receptors. Establishment of a general strategy to effectively combine a signal transducer with a receptor is still a demanding request to realize biosensors in a tailored manner.

Aptamer-based biosensors are fraught with challenges in terms of practical applications in cells, although this strategy is also a potentially promising approach for visualizing intracellular molecules. This is largely attributed to the difficulty in construction of aptamers with appropriate affinity and selectivity comparable to the native receptor protein, and the inherent lability of RNA molecules in the intracellular condition. These limitations would be overcome by the improvement of the selection and evolution technique, and the construction of signaling aptamers resistance to the cellular degradation activity.

## References

[1] Giepmans B.N., Adams S.R., Ellisman M.H., Tsien R.Y. (2006). The Fluorescent Toolbox for Assessing Protein Location and Function. Science.

[2] Johnsson N., Johnsson K. (2007). Chemical Tools for Biomolecular Imaging. ACS Chem. Biol.

[3] Rao J., Dragulescu-Andrasi A., Yao H. (2007). Fluorescence Imaging *in vivo*: Recent Advances. Curr. Opin. Biotechnol.

[4] Johnsson K. (2009). Visualizing Biochemical Activities in Living Cells. Nat. Chem. Biol.

[5] Wang H., Nakata E., Hamachi I. (2009). Recent Progress in Strategies for the Creation of Protein-Based Fluorescent Biosensors. ChemBioChem.

[6] de Silva A.P., Gunaratne H.Q., Gunnlaugsson T., Huxley A.J., McCoy C.P., Rademacher J.T., Rice T.E. (1997). Signaling Recognition Events with Fluorescent Sensors and Switches. Chem. Rev.

[7] Johnson I. (1998). Fluorescent Probes for Living Cells. Histochem. J.

[8] Terai T., Nagano T. (2008). Fluorescent Probes for Bioimaging Applications. Curr. Opin. Chem. Biol.

[9] Domaille D.W., Que E.L., Chang C.J. (2008). Synthetic Fluorescent Sensors for Studying the Cell Biology of Metals. Nat. Chem. Biol.

[10] Cao H., Heagy M.D. (2004). Fluorescent Chemosensors for Carbohydrates: a Decade's Worth of Bright Spies for Saccharides in Review. J. Fluoresc.

[11] Gomes A., Fernandes E., Lima J.L. (2006). Use of Fluorescence Probes for Detection of Reactive Nitrogen Species: a Review. J. Fluoresc.

[12] Soh N. (2006). Recent Advances in Fluorescent Probes for the Detection of Reactive Oxygen Species. Anal. Bioanal. Chem.

[13] Nolan E.M., Lippard S.J. (2009). Small-Molecule Fluorescent Sensors for Investigating Zinc Metalloneurochemistry. Acc. Chem. Res.

[14] Hellinga H.W., Marvin J.S. (1998). Protein Engineering and the Development of Generic Biosensors. Trends Biotechnol.

[15] Liu J., Cao Z., Lu Y. (2009). Functional Nucleic Acid Sensors. Chem. Rev.

[16] Stojanovic M.N., Kolpashchikov D.M. (2004). Modular Aptameric Sensors. J. Am. Chem. Soc.

[17] Hagihara M., Fukuda M., Hasegawa T., Morii T. (2006). A Modular Strategy for Tailoring Fluorescent Biosensors from Ribonucleopeptide Complexes. J. Am. Chem. Soc.

[18] Shimomura O., Johnson F.H., Saiga Y. (1962). Extraction, Purification and Properties of Aequorin, a Bioluminescent Protein from the Luminous Hydromedusan, Aequorea. J. Cell Comp. Physiol.

[19] Chalfie M., Tu Y., Euskirchen G., Ward W.W., Prasher D.C. (1994). Green Fluorescent Protein as a Marker for Gene Expression. Science.

[20] Lippincott-Schwartz J., Snapp E., Kenworthy A. (2001). Studying Protein Dynamics in Living Cells. Nat. Rev. Mol. Cell. Biol.

[21] Heim R., Prasher D.C., Tsien R.Y. (1994). Wavelength Mutations and Posttranslational Autoxidation of Green Fluorescent Protein. Proc. Natl. Acad. Sci. USA.

[22] Remington S.J. (2006). Fluorescent Proteins: Maturation, Photochemistry and Photophysics. Curr. Opin. Struct. Biol.

[23] Zhang J., Campbell R.E., Ting A.Y., Tsien R.Y. (2002). Creating New Fluorescent Probes for Cell Biology. Nat. Rev. Mol. Cell Biol.

[24] Kneen M., Farinas J., Li Y., Verkman A.S. (1998). Green Fluorescent Protein as a Noninvasive Intracellular pH Indicator. Biophys. J.

[25] Llopis J., McCaffery J.M., Miyawaki A., Farquhar M.G., Tsien R.Y. (1998). Measurement of Cytosolic, Mitochondrial, and Golgi pH in Single Living Cells with Green Fluorescent Proteins. Proc. Natl. Acad. Sci. USA.

[26] Miesenbock G., De Angelis D.A., Rothman J.E. (1998). Visualizing Secretion and Synaptic Transmission with pH-Sensitive Green Fluorescent Proteins. Nature.

[27] Wachter R.M., Yarbrough D., Kallio K., Remington S.J. (2000). Crystallographic and Energetic Analysis of Binding of Selected Anions to the Yellow Variants of Green Fluorescent Protein. J. Mol. Biol.

[28] Wachter R.M., Remington S.J. (1999). Sensitivity of the Yellow Variant of Green Fluorescent Protein to Halides and Nitrate. Curr. Biol.

[29] Jayaraman S., Haggie P., Wachter R.M., Remington S.J., Verkman A.S. (2000). Mechanism and Cellular Applications of a Green Fluorescent Protein-Based Halide Sensor. J. Biol. Chem.

[30] Chapleau R.R., Blomberg R., Ford P.C., Sagermann M. (2008). Design of a Highly Specific and Noninvasive Biosensor Suitable for Real-Time *in vivo* Imaging of Mercury (II) Uptake. Protein Sci.

[31] Barondeau D.P., Kassmann C.J., Tainer J.A., Getzoff E.D. (2002). Structural Chemistry of a Green Fluorescent Protein Zn Biosensor. J. Am. Chem. Soc.

[32] Mank M., Griesbeck O. (2008). Genetically Encoded Calcium Indicators. Chem. Rev.

[33] Garaschuk O., Griesbeck O., Konnerth A. (2007). Troponin C-Based Biosensors: a New Family of Genetically Encoded Indicators for *in vivo* Calcium Imaging in the Nervous System. Cell Calcium.

[34] Palmer A.E., Jin C., Reed J.C., Tsien R.Y. (2004). Bcl-2-Mediated Alterations in Endoplasmic Reticulum Ca^2+^ Analyzed with an Improved Genetically Encoded Fluorescent Sensor. Proc. Natl. Acad. Sci. USA.

[35] Palmer A.E., Giacomello M., Kortemme T., Hires S.A., Lev-Ram V., Baker D., Tsien R.Y. (2006). Ca^2+^ Indicators Based on Computationally Redesigned Calmodulin-Peptide Pairs. Chem. Biol.

[36] Miyawaki A., Llopis J., Heim R., McCaffery J.M., Adams J.A., Ikura M., Tsien R.Y. (1997). Fluorescent Indicators for Ca^2+^ Based on Green Fluorescent Proteins and Calmodulin. Nature.

[37] Mank M., Reiff D.F., Heim N., Friedrich M.W., Borst A., Griesbeck O. (2006). A FRET-Based Calcium Biosensor with Fast Signal Kinetics and High Fluorescence Change. Biophys. J.

[38] Nakai J., Ohkura M., Imoto K. (2001). A High Signal-to-Noise Ca^2+^ Probe Composed of a Single Green Fluorescent Protein. Nat. Biotechnol.

[39] Souslova E.A., Belousov V.V., Lock J.G., Stromblad S., Kasparov S., Bolshakov A.P., Pinelis V.G., Labas Y.A., Lukyanov S., Mayr L.M., Chudakov D.M. (2007). Single Fluorescent Protein-Based Ca^2+^ Sensors with Increased Dynamic Range. BMC Biotechnol.

[40] Baird G.S., Zacharias D.A., Tsien R.Y. (1999). Circular Permutation and Receptor Insertion within Green Fluorescent Proteins. Proc. Natl. Acad. Sci. USA.

[41] Romoser V.A., Hinkle P.M., Persechini A. (1997). Detection in Living Cells of Ca^2+^-Dependent Changes in the Fluorescence Emission of an Indicator Composed of Two Green Fluorescent Protein Variants Linked by a Calmodulin-Binding Sequence. A New Class of Fluorescent Indicators. J. Biol. Chem.

[42] Truong K., Sawano A., Mizuno H., Hama H., Tong K.I., Mal T.K., Miyawaki A., Ikura M. (2001). FRET-Based *in vivo* Ca^2+^ Imaging by a New Calmodulin-GFP Fusion Molecule. Nat. Struct. Biol.

[43] Nagai T., Sawano A., Park E.S., Miyawaki A. (2001). Circularly Permuted Green Fluorescent Proteins Engineered to Sense Ca^2+^. Proc Natl. Acad. Sci. USA.

[44] Imamura H., Nhat K.P., Togawa H., Saito K., Iino R., Kato-Yamada Y., Nagai T., Noji H. (2009). Visualization of ATP Levels inside Single Living Cells with Fluorescence Resonance Energy Transfer-Based Genetically Encoded Indicators. Proc. Natl. Acad. Sci. USA.

[45] DiPilato L.M., Cheng X., Zhang J. (2004). Fluorescent Indicators of cAMP and Epac Activation Reveal Differential Dynamics of cAMP Signaling within Discrete Subcellular Compartments. Proc. Natl. Acad. Sci. USA.

[46] Nikolaev V.O., Bunemann M., Schmitteckert E., Lohse M.J., Engelhardt S. (2006). Cyclic AMP Imaging in Adult Cardiac Myocytes Reveals Far-Reaching β1-Adrenergic but Locally Confined β2-Adrenergic Receptor-Mediated Signaling. Circ. Res.

[47] Zaccolo M., De Giorgi F., Cho C.Y., Feng L., Knapp T., Negulescu P.A., Taylor S.S., Tsien R.Y., Pozzan T. (2000). A Genetically Encoded, Fluorescent Indicator for Cyclic AMP in Living Cells. Nat. Cell Biol.

[48] Zaccolo M., Pozzan T. (2002). Discrete Microdomains with High Concentration of cAMP in Stimulated Rat Neonatal Cardiac Myocytes. Science.

[49] Sato M., Nakajima T., Goto M., Umezawa Y. (2006). Cell-Based Indicator to Visualize Picomolar Dynamics of Nitric Oxide Release from Living Cells. Anal. Chem.

[50] Honda A., Adams S.R., Sawyer C.L., Lev-Ram V., Tsien R.Y., Dostmann W.R. (2001). Spatiotemporal Dynamics of Guanosine 3′,5′-Cyclic Monophosphate Revealed by a Genetically Encoded, Fluorescent Indicator. Proc. Natl. Acad. Sci. USA.

[51] Nikolaev V.O., Gambaryan S., Lohse M.J. (2006). Fluorescent Sensors for Rapid Monitoring of Intracellular cGMP. Nat. Methods.

[52] Kaper T., Looger L.L., Takanaga H., Platten M., Steinman L., Frommer W.B. (2007). Nanosensor Detection of an Immunoregulatory Tryptophan Influx/Kynurenine Efflux Cycle. PLoS Biol.

[53] Okumoto S., Looger L.L., Micheva K.D., Reimer R.J., Smith S.J., Frommer W.B. (2005). Detection of Glutamate Release from Neurons by Genetically Encoded Surface-Displayed FRET Nanosensors. Proc. Natl. Acad. Sci. USA.

[54] Tsien R.Y. (2005). Building and Breeding Molecules to Spy on Cells and Tumors. FEBS Lett.

[55] Hirose K., Kadowaki S., Tanabe M., Takeshima H., Iino M. (1999). Spatiotemporal Dynamics of Inositol 1,4,5-Trisphosphate That Underlies Complex Ca^2+^ Mobilization Patterns. Science.

[56] Sakaguchi R., Endoh T., Yamamoto S., Tainaka K., Sugimoto K., Fujieda N., Kiyonaka S., Mori Y., Morii T. (2009). A Single Circularly Permuted GFP Sensor for Inositol-1,3,4,5-Tetrakisphosphate Based on a Split PH Domain. Bioorg. Med. Chem.

[57] Sapsford K.E., Berti L., Medintz I.L. (2006). Materials for Fluorescence Resonance Energy Transfer Analysis: Beyond Traditional Donor-Acceptor Combinations. Angew. Chem. Int. Ed.

[58] Piston D.W., Kremers G.J. (2007). Fluorescent Protein FRET: The Good, the Bad and the Ugly. Trends Biochem. Sci.

[59] Ohashi T., Galiacy S.D., Briscoe G., Erickson H.P. (2007). An Experimental Study of GFP-Based FRET, with Application to Intrinsically Unstructured Proteins. Protein Sci.

[60] Souslova E.A., Chudakov D.M. (2007). Genetically Encoded Intracellular Sensors Based on Fluorescent Proteins. Biochemistry (Mosc).

[61] VanEngelenburg S.B., Palmer A.E. (2008). Fluorescent Biosensors of Protein Function. Curr. Opin. Chem. Biol.

[62] Carlson H.J., Campbell R.E. (2009). Genetically Encoded FRET-Based Biosensors for Multiparameter Fluorescence Imaging. Curr. Opin. Biotechnol.

[63] Brun M.A., Tan K.T., Nakata E., Hinner M.J., Johnsson K. (2009). Semisynthetic Fluorescent Sensor Proteins Based on Self-Labeling Protein Tags. J. Am. Chem. Soc.

[64] Keppler A., Gendreizig S., Gronemeyer T., Pick H., Vogel H., Johnsson K. (2003). A General Method for the Covalent Labeling of Fusion Proteins with Small Molecules *in vivo*. Nat. Biotechnol.

[65] Nausch L.W., Ledoux J., Bonev A.D., Nelson M.T., Dostmann W.R. (2008). Differential Patterning of cGMP in Vascular Smooth Muscle Cells Revealed by Single GFP-Linked Biosensors. Proc. Natl. Acad. Sci. USA.

[66] Belousov V.V., Fradkov A.F., Lukyanov K.A., Staroverov D.B., Shakhbazov K.S., Terskikh A.V., Lukyanov S. (2006). Genetically Encoded Fluorescent Indicator for Intracellular Hydrogen Peroxide. Nat. Methods.

[67] Dooley C.T., Dore T.M., Hanson G.T., Jackson W.C., Remington S.J., Tsien R.Y. (2004). Imaging Dynamic Redox Changes in Mammalian Cells with Green Fluorescent Protein Indicators. J. Biol. Chem.

[68] Mizuno T., Murao K., Tanabe Y., Oda M., Tanaka T. (2007). Metal-Ion-Dependent GFP Emission *in vivo* by Combining a Circularly Permutated Green Fluorescent Protein with an Engineered Metal-Ion-Binding Coiled-Coil. J. Am. Chem. Soc.

[69] Ghosh I., Hamilton A.D., Regan L. (2000). Antiparallel Leucine Zipper-Directed Protein Reassembly: Application to the Green Fluorescent Protein. J. Am. Chem. Soc.

[70] Stains C.I., Porter J.R., Ooi A.T., Segal D.J., Ghosh I. (2005). DNA Sequence-Enabled Reassembly of the Green Fluorescent Protein. J. Am. Chem. Soc.

[71] Demidov V.V., Dokholyan N.V., Witte-Hoffmann C., Chalasani P., Yiu H.W., Ding F., Yu Y., Cantor C.R., Broude N.E. (2006). Fast Complementation of Split Fluorescent Protein Triggered by DNA Hybridization. Proc. Natl. Acad. Sci. USA.

[72] Stains C.I., Furman J.L., Segal D.J., Ghosh I. (2006). Site-Specific Detection of DNA Methylation Utilizing mCpG-SEER. J. Am. Chem. Soc.

[73] Ozawa T., Natori Y., Sato M., Umezawa Y. (2007). Imaging Dynamics of Endogenous Mitochondrial RNA in Single Living Cells. Nat. Methods.

[74] Valencia-Burton M., McCullough R.M., Cantor C.R., Broude N.E. (2007). RNA Visualization in Live Bacterial Cells Using Fluorescent Protein Complementation. Nat. Methods.

[75] Marrero M.B., Schieffer B., Paxton W.G., Schieffer E., Bernstein K.E. (1995). Electroporation of pp60^c-src^ Antibodies Inhibits the Angiotensin II Activation of Phospholipase C-γ1 in Rat Aortic Smooth Muscle Cells. J. Biol. Chem.

[76] Fenton M., Bone N., Sinclair A.J. (1998). The Efficient and Rapid Import of a Peptide into Primary B and T Lymphocytes and a Lymphoblastoid Cell Line. J. Immunol. Methods.

[77] Sakaguchi R., Tainaka K., Shimada N., Nakano S., Inoue M., Kiyonaka S., Mori Y., Morii T. (2009). An *in vivo* Fluorescent Sensor Reveals Intracellular Ins(1,3,4,5)P_4_ Dynamics in Single Cells. Angew. Chem. Int. Ed.

[78] Zelphati O., Wang Y., Kitada S., Reed J.C., Felgner P.L., Corbeil J. (2001). Intracellular Delivery of Proteins with a New Lipid-Mediated Delivery System. J. Biol. Chem.

[79] Zheng X., Lundberg M., Karlsson A., Johansson M. (2003). Lipid-Mediated Protein Delivery of Suicide Nucleoside Kinases. Cancer Res.

[80] Abarzua P., LoSardo J.E., Gubler M.L., Neri A. (1995). Microinjection of Monoclonal Antibody PAb421 into Human SW480 Colorectal Carcinoma Cells Restores the Transcription Activation Function to Mutant p53. Cancer Res.

[81] Wadia J.S., Dowdy S.F. (2005). Transmembrane Delivery of Protein and Peptide Drugs by TAT-Mediated Transduction in the Treatment of Cancer. Adv. Drug. Deliv. Rev.

[82] Sugimoto K., Nishida M., Otsuka M., Makino K., Ohkubo K., Mori Y., Morii T. (2004). Novel Real-Time Sensors to Quantitatively Assess *in vivo* Inositol 1,4,5-Trisphosphate Production in Intact Cells. Chem. Biol.

[83] Dwyer M.A., Hellinga H.W. (2004). Periplasmic Binding Proteins: A Versatile Superfamily for Protein Engineering. Curr. Opin. Struct. Biol.

[84] Marvin J.S., Corcoran E.E., Hattangadi N.A., Zhang J.V., Gere S.A., Hellinga H.W. (1997). The Rational Design of Allosteric Interactions in a Monomeric Protein and Its Applications to the Construction of Biosensors. Proc. Natl. Acad. Sci. USA.

[85] Marvin J.S., Hellinga H.W. (1998). Engineering Biosensors by Introducing Fluorescent Allosteric Signal Transducers: Construction of a Novel Glucose Sensor. J. Am. Chem. Soc.

[86] de Lorimier R.M., Smith J.J., Dwyer M.A., Looger L.L., Sali K.M., Paavola C.D., Rizk S.S., Sadigov S., Conrad D.W., Loew L., Hellinga H.W. (2002). Construction of a Fluorescent Biosensor Family. Protein Sci.

[87] Marvin J.S., Hellinga H.W. (2001). Conversion of a Maltose Receptor into a Zinc Biosensor by Computational Design. Proc. Natl. Acad. Sci. USA.

[88] Dwyer M.A., Looger L.L., Hellinga H.W. (2003). Computational Design of a Zn^2+^ Receptor That Controls Bacterial Gene Expression. Proc. Natl. Acad. Sci. USA.

[89] Looger L.L., Dwyer M.A., Smith J.J., Hellinga H.W. (2003). Computational Design of Receptor and Sensor Proteins with Novel Functions. Nature.

[90] Allert M., Rizk S.S., Looger L.L., Hellinga H.W. (2004). Computational Design of Receptors for an Organophosphate Surrogate of the Nerve Agent Soman. Proc. Natl. Acad. Sci. USA.

[91] Schreier B., Stumpp C., Wiesner S., Hocker B. (2009). Computational Design of Ligand Binding Is Not a Solved Problem. Proc. Natl. Acad. Sci. USA.

[92] Telmer P.G., Shilton B.H. (2005). Structural Studies of an Engineered Zinc Biosensor Reveal an Unanticipated Mode of Zinc Binding. J. Mol. Biol.

[93] Brune M., Hunter J.L., Corrie J.E., Webb M.R. (1994). Direct, Real-Time Measurement of Rapid Inorganic Phosphate Release Using a Novel Fluorescent Probe and Its Application to Actomyosin Subfragment 1 ATPase. Biochemistry.

[94] Gilardi G., Zhou L.Q., Hibbert L., Cass A.E. (1994). Engineering the Maltose Binding Protein for Reagentless Fluorescence Sensing. Anal. Chem.

[95] Brune M., Hunter J.L., Howell S.A., Martin S.R., Hazlett T.L., Corrie J.E., Webb M.R. (1998). Mechanism of Inorganic Phosphate Interaction with Phosphate Binding Protein from *Escherichia coli.*. Biochemistry.

[96] Hirshberg M., Henrick K., Haire L.L., Vasisht N., Brune M., Corrie J.E., Webb M.R. (1998). Crystal Structure of Phosphate Binding Protein Labeled with a Coumarin Fluorophore, a Probe for Inorganic Phosphate. Biochemistry.

[97] Gilbert S.P., Webb M.R., Brune M., Johnson K.A. (1995). Pathway of Processive ATP Hydrolysis by Kinesin. Nature.

[98] Morii T., Sugimoto K., Makino K., Otsuka M., Imoto K., Mori Y. (2002). A New Fluorescent Biosensor for Inositol Trisphosphate. J. Am. Chem. Soc.

[99] Nishida M., Sugimoto K., Hara Y., Mori E., Morii T., Kurosaki T., Mori Y. (2003). Amplification of Receptor Signaling by Ca^2+^ Entry-Mediated Translocation and Activation of PLCγ2 in B Lymphocytes. EMBO J.

[100] Chan P.H., Liu H.B., Chen Y.W., Chan K.C., Tsang C.W., Leung Y.C., Wong K.Y. (2004). Rational Design of a Novel Fluorescent Biosensor for β-Lactam Antibiotics from a Class a β-Lactamase. J. Am. Chem. Soc.

[101] Chan P.H., So P.K., Ma D.L., Zhao Y., Lai T.S., Chung W.H., Chan K.C., Yiu K.F., Chan H.W., Siu F.M., Tsang C.W., Leung Y.C., Wong K.Y. (2008). Fluorophore-Labeled β-Lactamase as a Biosensor for β-Lactam Antibiotics: A Study of the Biosensing Process. J. Am. Chem. Soc.

[102] Wang L., Schultz P.G. (2005). Expanding the Genetic Code. Angew. Chem. Int. Ed.

[103] Xie J., Schultz P.G. (2006). A Chemical Toolkit for Proteins—an Expanded Genetic Code. Nat. Rev. Mol. Cell Biol.

[104] Hohsaka T., Sisido M. (2002). Incorporation of Non-Natural Amino Acids into Proteins. Curr. Opin. Chem. Biol.

[105] Anderson R.D., Zhou J., Hecht S.M. (2002). Fluorescence Resonance Energy Transfer between Unnatural Amino Acids in a Structurally Modified Dihydrofolate Reductase. J. Am. Chem. Soc.

[106] Taki M., Hohsaka T., Murakami H., Taira K., Sisido M. (2002). Position-Specific Incorporation of a Fluorophore-Quencher Pair into a Single Streptavidin through Orthogonal Four-Base Codon/Anticodon Pairs. J. Am. Chem. Soc.

[107] Kajihara D., Abe R., Iijima I., Komiyama C., Sisido M., Hohsaka T. (2006). FRET Analysis of Protein Conformational Change through Position-Specific Incorporation of Fluorescent Amino Acids. Nat. Methods.

[108] Nakata E., Tsukiji S., Hamachi I. (2007). Development of New Methods to Introduce Unnatural Functional Molecules into Native Proteins for Protein Engineering. Bull. Chem. Soc. Jpn.

[109] Hamachi I., Nagase T., Shinkai S. (2000). A General Semisynthetic Method for Fluorescent Saccharide-Biosensors Based on a Lectin. J. Am. Chem. Soc.

[110] Nakata E., Nagase T., Shinkai S., Hamachi I. (2004). Coupling a Natural Receptor Protein with an Artificial Receptor to Afford a Semisynthetic Fluorescent Biosensor. J. Am. Chem. Soc.

[111] Koshi Y., Nakata E., Hamachi I. (2005). Luminescent Saccharide Biosensor by Using Lanthanide-Bound Lectin Labeled with Fluorescein. ChemBioChem.

[112] Nakata E., Koshi Y., Koga E., Katayama Y., Hamachi I. (2005). Double-Modification of Lectin Using Two Distinct Chemistries for Fluorescent Ratiometric Sensing and Imaging Saccharides in Test Tube or in Cell. J. Am. Chem. Soc.

[113] Nakata E., Wang H., Hamachi I. (2008). Ratiometric Fluorescent Biosensor for Real-Time and Label-Free Monitoring of Fine Saccharide Metabolic Pathways. ChemBioChem.

[114] Tsukiji S., Miyagawa M., Takaoka Y., Tamura T., Hamachi I. (2009). Ligand-Directed Tosyl Chemistry for Protein Labeling *in vivo*. Nat. Chem. Biol.

[115] Tsukiji S., Wang H., Miyagawa M., Tamura T., Takaoka Y., Hamachi I. (2009). Quenched Ligand-Directed Tosylate Reagents for One-Step Construction of Turn-on Fluorescent Biosensors. J. Am. Chem. Soc.

[116] Ellington A.D. (1994). RNA Selection. Aptamers Achieve the Desired Recognition. Curr. Biol.

[117] Ellington A.D., Szostak J.W. (1990). *In vitro* Selection of RNA Molecules That Bind Specific Ligands. Nature.

[118] Gold L., Polisky B., Uhlenbeck O., Yarus M. (1995). Diversity of Oligonucleotide Functions. Annu. Rev. Biochem.

[119] Osborne S.E., Ellington A.D. (1997). Nucleic Acid Selection and the Challenge of Combinatorial Chemistry. Chem. Rev.

[120] Wilson D.S., Szostak J.W. (1999). *In vitro* Selection of Functional Nucleic Acids. Annu. Rev. Biochem.

[121] Westhof E., Patel D.J. (1997). Nucleic Acids. From Self-Assembly to Induced-Fit Recognition. Curr. Opin. Struct. Biol.

[122] Mok W., Li Y. (2008). Recent Progress in Nucleic Acid Aptamer-Based Biosensors and Bioassays. Sensors.

[123] Jhaveri S.D., Kirby R., Conrad R., Maglott E.J., Bowser M., Kennedy R.T., Glick G., Ellington A.D. (2000). Designed Signaling Aptamers That Transduce Molecular Recognition to Changes in Fluorescence Intensity. J. Am. Chem. Soc.

[124] Jhaveri S., Rajendran M., Ellington A.D. (2000). *In vitro* Selection of Signaling Aptamers. Nat. Biotechnol.

[125] Yamana K., Ohtani Y., Nakano H., Saito I. (2003). Bis-Pyrene Labeled DNA Aptamer as an Intelligent Fluorescent Biosensor. Bioorg. Med. Chem. Lett.

[126] Chamberlin S.I., Merino E.J., Weeks K.M. (2002). Catalysis of Amide Synthesis by RNA Phosphodiester and Hydroxyl Groups. Proc. Natl. Acad. Sci. USA.

[127] Merino E.J., Wilkinson K.A., Coughlan J.L., Weeks K.M. (2005). RNA Structure Analysis at Single Nucleotide Resolution by Selective 2′-Hydroxyl Acylation and Primer Extension (SHAPE). J. Am. Chem. Soc.

[128] Kamekawa N., Shimomura Y., Nakamura M., Yamana K. (2006). Pyrene-Modified DNA Aptamer as a Fluorescent Biosensor with High Affinity and Specificity for ATP Sensing. Chem. Lett.

[129] Merino E.J., Weeks K.M. (2005). Facile Conversion of Aptamers into Sensors Using a 2′-Ribose-Linked Fluorophore. J. Am. Chem. Soc.

[130] Katilius E., Katiliene Z., Woodbury N.W. (2006). Signaling Aptamers Created Using Fluorescent Nucleotide Analogues. Anal. Chem.

[131] Ueyama H., Takagi M., Takenaka S. (2002). A Novel Potassium Sensing in Aqueous Media with a Synthetic Oligonucleotide Derivative. Fluorescence Resonance Energy Transfer Associated with Guanine Quartet-Potassium Ion Complex Formation. J. Am. Chem. Soc.

[132] Nagatoishi S., Nojima T., Galezowska E., Juskowiak B., Takenaka S. (2006). G Quadruplex-Based FRET Probes with the Thrombin-Binding Aptamer (TBA) Sequence Designed for the Efficient Fluorometric Detection of the Potassium Ion. ChemBioChem.

[133] Ono A., Togashi H. (2004). Highly Selective Oligonucleotide-Based Sensor for Mercury(II) in Aqueous Solutions. Angew. Chem. Int. Ed.

[134] Stojanovic M.N., de Prada P., Landry D.W. (2001). Aptamer-Based Folding Fluorescent Sensor for Cocaine. J. Am. Chem. Soc.

[135] Ozaki H., Nishihira A., Wakabayashi M., Kuwahara M., Sawai H. (2006). Biomolecular Sensor Based on Fluorescence-Labeled Aptamer. Bioorg. Med. Chem. Lett.

[136] Urata H., Nomura K., Wada S., Akagi M. (2007). Fluorescent-Labeled Single-Strand ATP Aptamer DNA: Chemo- and Enantio-Selectivity in Sensing Adenosine. Biochem. Biophys. Res. Commun.

[137] Li J.J., Fang X., Schuster S.M., Tan W. (2000). Molecular Beacons: A Novel Approach to Detect Protein - DNA Interactions. Angew. Chem. Int. Ed.

[138] Hamaguchi N., Ellington A., Stanton M. (2001). Aptamer Beacons for the Direct Detection of Proteins. Anal. Biochem.

[139] Yang C.J., Jockusch S., Vicens M., Turro N.J., Tan W. (2005). Light-Switching Excimer Probes for Rapid Protein Monitoring in Complex Biological Fluids. Proc. Natl. Acad. Sci. USA.

[140] Yamamoto R., Baba T., Kumar P.K. (2000). Molecular Beacon Aptamer Fluoresces in the Presence of Tat Protein of HIV-1. Genes Cells.

[141] Stojanovic M.N., de Prada P., Landry D.W. (2000). Fluorescent Sensors Based on Aptamer Self-Assembly. J. Am. Chem. Soc.

[142] Nutiu R., Li Y. (2003). Structure-Switching Signaling Aptamers. J. Am. Chem. Soc.

[143] Nutiu R., Li Y. (2005). A DNA-Protein Nanoengine For “On-Demand” Release and Precise Delivery of Molecules. Angew. Chem. Int. Ed.

[144] Morii T., Hagihara M., Sato S., Makino K. (2002). *In vitro* Selection of ATP-Binding Receptors Using a Ribonucleopeptide Complex. J. Am. Chem. Soc.

[145] Fukuda M., Hayashi H., Hasegawa T., Morii T. (2009). Development of a Fluorescent Ribonucleopeptide Sensor for Histamine. Trans. Mat. Res. Soc. Jpn.

[146] Hasegawa T., Ohkubo K., Yoshikawa S., Morii T. (2005). A Ribonucleopeptide Receptor Targets Phosphotyrosine. J. Surf. Sci. Nanotech.

[147] Hasegawa T., Hagihara M., Fukuda M., Nakano S., Fujieda N., Morii T. (2008). Context-Dependent Fluorescence Detection of a Phosphorylated Tyrosine Residue by a Ribonucleopeptide. J. Am. Chem. Soc.

